# Implementing mhGAP training to strengthen existing services for an internally displaced population in Pakistan

**DOI:** 10.1017/gmh.2017.1

**Published:** 2017-04-03

**Authors:** A. Humayun, I. Haq, F. R. Khan, N. Azad, M. M. Khan, I. Weissbecker

**Affiliations:** 1 Meditrina Healthcare, Rawalpindi, Pakistan; 2 KRL Hospital, Islamabad, Pakistan; 3 Al-Nafees Medical College, Isra University, Islamabad, Pakistan; 4 Foundation University Medical College, Rawalpindi, Pakistan; 5 Aga Khan University, Karachi, Pakistan; 6 Global Mental Health &Psychosocial Advisor International Medical Corps, Washington, DC, USA

**Keywords:** Internally displaced people, mental health, mhGAP training, Pakistan, psychosocial support

## Abstract

**Background.:**

In 2014, over a million people were internally displaced after the launch of a military operation in North Waziristan, a tribal region on Pakistan's side of the Durand Line. Despite security concerns and restrictions, a collaborative mental health and psychosocial support initiative was undertaken in the district of Bannu. Monthly mental health camps were conducted for a period of 6 months by a multidisciplinary mental health team. The initiative also helped to assess mental health needs and plan training for primary care staff to strengthen existing resources.

**Methods.:**

As part of this initiative, Mental Health Gap Action Programme (mhGAP) training was conducted for physicians and psychosocial staff in the affected district. This marked the first instance of implementing these guidelines in Pakistan following a humanitarian crisis. This paper describes the training process including the adaptation of the mhGAP curriculum, training of trainers, training workshops for primary care staff and an analysis of results of pre- and post-testing of their knowledge about common mental disorders using a 25-item questionnaire.

**Results.:**

The gaps in knowledge of primary care physicians in recognizing and managing common mental disorders were clearly identified. The mean pre- and post-test scores of the participants were 15.43, 62% (*p* value 0.000, s.d. 4.05) and 19.48, 78% (*p* value 0.000, s.d. 3.13) respectively, which showed significant improvement.

**Conclusions.:**

Despite the challenges of a humanitarian crisis, mhGAP guidelines can be successfully implemented to train primary care physicians in in low- and middle-income countries such as Pakistan. However, the dearth of primary care resources can hinder the complete integration of mental health services into primary healthcare.

## Background

Recent estimates show that the global burden of mental illness is a serious public health concern, accounting for 32·4% of years lived with disability and 13% of disability-adjusted life-years (DALYs) (Vigo *et al.* 2016). Despite these estimates, resources needed to address the burden are inadequate (Saxena *et al*. 2007). More than 75% of the treatment gap for mental disorders exists in low- and middle-income countries (LMICs) where four out of five people with mental illness do not receive effective treatment (Dua *et al.* 2011). This treatment gap persists even though effective low-cost treatments can be provided in primary healthcare settings (WHO, 2008; Eaton, 2011). A major barrier to address this gap in LMIC is the scarcity and unequal distribution of specialist mental health professionals (Saraceno *et al.* 2007). There is an estimated shortage of 1.18 million mental health workers in LMICs alone (WHO, 2011). This huge disparity can be addressed through the training of primary care staff to recognize and treat mental disorders (Patel, 2008). There have been some successful trainings aimed at integrating mental health into primary care in countries such as Afghanistan (Ventevogel *et al.* 2012), Sri Lanka (Jenkins, 2012), Pakistan and Jordan (Budosan, 2011), Lebanon (Hijazi *et al.* 2011) and Iraq (Sadiq, 2011).

In 2010, the WHO launched the Mental Health Gap Action Programme (mhGAP), to assist LMICs to scale up mental health services (WHO, 2010). The mhGAP-Intervention Guide (mhGAP-IG) offers guidelines to enable non-specialists in primary healthcare to detect and treat priority disorders, and make appropriate referrals to the next level of care (Dua *et al.* 2011). Since then, there have been encouraging reports of mhGAP training in several resource-limited settings (Hijazi *et al.* 2011; Hussain & Hughes, 2013; Andrea, 2014; Gureje *et al.* 2015).

Like most LMICs, Pakistan faces an overwhelming challenge of scarcity of mental health resources with only 342 psychiatrists in a population of 182 million (0.20 per 100 000 population) (WHO, 2009). Glaring gaps are reported in the capacity of primary care physicians to address mental disorders in Pakistan (Naqvi *et al.* 2012). There have been sporadic community mental health initiatives to train non-specialist health workers (Ali *et al.* 2015). However, the need to strengthen the capacity of primary care staff has already been identified as a priority intervention in the province of Khyber Pakhtunkhwa (KP), which has been facing ongoing geo-political conflict for decades (Budosan & Aziz, 2009; Shah *et al.* 2014). In addition, some attempts to train primary care staff have also been made in the province, but to the best of our knowledge no effort has been made to implement mhGAP-IG for training primary care physicians in Pakistan.

In June 2014, a military operation in North Waziristan displaced over a million people into the district of Bannu (KPK), where mental health services were limited to a single psychiatrist. In view of the emergency situation, a mental health and psychosocial support (MHPSS) initiative was launched (Humayun *et al.* 2016). Despite severely restricted conditions, a monthly mental health camp was held for 6 months to strengthen the local mental health services. The camps also provided an opportunity to assess needs and train healthcare and psychosocial staff working in the district. As part of this initiative, International Medical Corps (IMC) contracted the first author (AH) to conduct mhGAP training for primary care staff. The training was conducted in collaboration with the Pakistan Army Field Hospital, which administered the main teaching hospital in Bannu & the District Health Office (DHO).

In this paper, we describe the training process, including the curriculum development, training of trainers (ToT), training workshops conducted, provision of hands-on supervision in the camps and the establishment of a referral system.

## Method

Joint consultations with the DHO were held over a period of 2 months before the start of the actual trainings. The camps were instrumental in advocacy and need assessment including the psychosocial context of the displaced population, the presentations of common mental disorders and the local healthcare system. The mhGAP-IG was used as a key reference and adapted to local mental health needs and the competence of primary care staff (WHO, 2010). Despite the fact that the mhGAP-IG presents comprehensive clinical protocols and algorithms, we found the interface of the guide quite complex for our setting and had to simplify further. Most of the training was designed/conducted in Urdu (the national language) since the participants were not accustomed to the English language, even in clinical settings. In addition, interventions and indications for referral were clearly defined considering the existing healthcare system. Some specific changes in adapting

mhGAP are described below:

### (a) Developing a curriculum

Priority disorders identified from the camps included depression, adjustment disorders, intellectual disability, epilepsy, behavioral disorders and drug dependence (Humayun *et al.* 2016). In total, 28% of the cases assessed at these camps were under the age of 18. Nearly a third of these cases (children) presented with intellectual disability and another third with behavioral disorders. Based on our experience and appropriate adaptation of mhGAP guidelines, we developed six modules: stress-related disorders; depression; psychosis; child & adolescent mental health (includes learning disability); epilepsy & drug dependence. The detailed curriculum is shown in Table 1.

**Table 1.** Curriculum of six modules

Modules	Hr 14	Objectives - The doctor should be able to:	Content outline	Methodology
1. Stress-related disorders	3	Recognize the impact of stress on mental and physical health Identify stress-related conditions/disorders Treat stress-related conditions/ disorders Refer appropriately	Effect of stress on mental and physical health Nature of stress-related conditions/disorders Introduction to general psychological interventions Management of common stress-related disorders Indicators to refer	Impact of stress on health – 20 min *Large group discussion +PPP* Clinical interview: a recent loss – 20 min *Role play* Presentation of stress-related disorders – 20 min *PowerPoint presentation* Pharmacological interventions – 20 min *Small group discussion* Non pharmacological interventions – 30 min *PowerPoint presentation* Grief: Presentation & management – 30 min *Large group discussion* Q & A – 10 min
2. Depression	3	Diagnose depression and assess severity Treat depression (counseling and pharmacological) Provide regular follow up Refer appropriately	Signs and symptoms Difference between mild, moderate, severe depression Assessment guidelines Non-pharmacological interventions (counseling) for depression Antidepressants (Prescribing, monitoring and terminating treatment) Indications to refer	Introduction to depression – 20 min (Nature, prevalence, morbidity & mortality) *Large group discussion & PPP* Clinical interview – 20 min *Role play* Demonstrating the severity – 20 min *Case scenarios* Investigations – 20 min (Causes, presentation and differential diagnoses) *Large group discussion* Non pharmacological treatment – 30 min *Small group discussion* Pharmacological treatment – 30 min Small group discussion & PPP Q & A – 10 min
3. Psychosis	2	Diagnose psychosis Initiate treatment Refer appropriately Monitor & follow up	Introduction to common psychotic disorders Common signs and symptoms Assessment guidelines Psychosocial and pharmacological interventions Indicators to monitor and refer	Common disorders – 30 min *Large group discussion +PPP* Recognising symptoms/signs – 30 min *Case scenarios* Clinical assessment – 20 min *PPP* Treatment, course & referral -30 min *PPP* Q & A – 10 min
4. CAMH & learning disability	2	Assess normal development Identify common childhood disorders Diagnose learning disability Guide parents for further management Refer appropriately	Common presentations in children and adolescents Assessment of a child with learning disability Outline of psychosocial and behavioral management Indications to refer	Child development & common clinical presentations – 30 min *Large group discussion* Classification of Learning disability & common causes – 10 min *PPP* Clinical assessment 30 min *Small group discussion* Family counseling/Psycho-education – 20 min *Role play* Behavioural management – 20 min *PPP* Q & A – 10 min
5. Epilepsy	2	Diagnose epilepsy Treat epilepsy Manage an emergency Refer appropriately	Assessment of epilepsy Anticonvulsants (dosage & monitoring treatment) Emergency management of epilepsy Indicator to refer	Types, prevalence & treatment gap – 30 min *Large group discussion* Clinical assessment/ investigations – 30 min *Small group discussion* 3.Treatment outline – 30 min *PPP* Counselling family – 20 min *Role play* Q&A 10 min
6. Drug dependence	2	Diagnose drug dependence and withdrawal Assess motivation to change Minimize harm and manage Refer appropriately	Common drugs of abuse Criteria for drug dependence and withdrawal Assessment including laboratory investigations Motivational interview Brief outline of interventions Indicators to refer	Introduction to concept – 20 min *Large group discussion* Clinical assessment – 20 min *PPP* Taking drug history – 20 min *Role play* Assessing motivation – 10 min *Video* Treatment options – 30 min *PPP* Q & A – 10 min

Our module on ‘stress-related disorders’ was adapted from WHO SPE-STRESS Intervention Guidelines (WHO, 2013a). We included the concept of stress and normal reactions to stress (this had not been part of undergraduate training of our target group). Based on our experience at the camps, the objective was to address stress-related conditions in general and not focus on specific categories such as acute stress, post-traumatic stress disorder, etc. We also included dissociative disorder, as it was highly relevant in this context, and laid an emphasis on common presentations of conversion reactions seen in the camps. Conducting the stress module at the outset helped participants rule out conditions related to stress (including grief) before embarking on a clinical diagnosis of depression.

The module on child & adolescent mental health (CAMH) was relatively difficult to prepare since we included information from three modules of mhGAP-IG. These were: (1) general framework of working with children; (2) mental retardation; and (3) behavioral disorders. Discussions were mainly centered on the behavioral management of children with mental health problems and the psycho-education of the parents of children with disability. From our experience at the camps, we highlighted the ‘Don'ts’ to avoid unnecessary investigations (especially electroencephalogram, computed tomography scan and magnetic resonance imaging) and psychotropic prescriptions in children.

In the module on psychosis, more emphasis was needed on symptom recognition than is suggested by the mhGAP guide. The module on drug dependence mainly focused on three drugs of abuse: opioids, cannabis and benzodiazepines. We omitted replacement therapy as it was not relevant to our context and instead emphasized on psychosocial aspects of management, including motivational counseling.

The hallmark of our training was the focus on a range of psychosocial interventions, for which we used the term ‘Counseling’ during training. These were highlighted in all modules and included psycho-education, behavioral activation, stress management, problem solving and the application of principles of behavioral therapy and supportive counseling (Table 1 shows individual psychological interventions in the curriculum). The clinical protocols for these psychological interventions were previously discussed by Humayun *et al.* (2016).

Where necessary, pharmacological guidelines were prepared by considering drugs commonly available in local market.

### (b) Training of trainers

Three psychiatrists (IH, FRK and NA) were identified as potential trainers from the team conducting the mental health camps. In addition to their clinical experience, personal attributes such as integrity, motivation & the ability to work beyond the traditional biological model were important considerations in their selection. The trainers were encouraged to take a proactive role in reviewing mhGAP training modules and adapt these to local needs (over a period of 4 weeks). The master trainer supervised this process as these guidelines were also used for treatments protocols offered in the camps. A final 1-day ToT Workshop focused on the rationale for integrating mental health in primary care and the content and methodology of mhGAP guidelines. The three trainers made formal presentations on each module. Two external reviewers were invited to give independent feedback regarding the objectives, course content, pedagogy and teaching skills of the trainers. Based on the modules and adapted from mhGAP guidelines, a training manual was prepared (Bannu Training Manual, 2014). This was also peer reviewed by senior mental health professionals in the country and by the Global Mental Health and Psychosocial Advisor for IMC.

### (c) Training workshops

A total of three 2-day training workshops were held over a period of 3 months (November 2014–January 2015). These were attended by 58 participants, of which 51 were doctors. These included primary care physicians (18); doctors from teaching hospital (11); doctors from secondary care facilities (14); medical administrators (3); and doctors from North Waziristan (tribal area of the displaced population) (5). In addition, the psychosocial staff of humanitarian agencies (7) working closely with the government healthcare facilities also participated.

The teaching sessions were interactive and included large and small group discussions, individual exercises & seminar presentations. The videos included in the mhGAP package were not applicable because of language/cultural barriers. Instead, role-play was used to demonstrate clinical skills.

### (d) Providing hands-on supervision

Monthly camps were held for a period of 6 months, where 785 cases were seen, by teams of specialists (including master trainer and trainers) and non-specialists (Humayun *et al*. 2016). Joint assessments were conducted on each case in 15–20 min consultations. Initially, the non-specialist staff observed, interpreted clinical information and helped by reinforcing instructions/advice. Later, they led the interviews, where specialists facilitated as required. In many cases, the non-specialists followed up cases in the community otherwise follow-ups were organized at the department of psychiatry. In addition to hands-on supervision, formal and informal case discussions were part of supervision. Over the course of the supervisions, the need for holistic assessments, psychosocial interventions and timely referrals was emphasized. Following the camps, the local psychiatrist continued to supervise primary care staff informally especially to follow up difficult cases that were once referred to the psychiatric facility.

### (e) Developing a referral system

The trainings also provided a forum for introducing and networking available mental health and psychosocial facilities. The hospital administration offered their support for referrals from primary care. A telephone helpline was also set up at the department of psychiatry for acute emergency referrals.

(For more information and images of these training sessions: http://www.meditrina.pk/index.php/trainings/mhgap/)

## Results

The knowledge about common mental disorders was assessed using a 25-item Questionnaire, adapted from mhGAP guidelines (Bannu Questionnaire, 2014). The test was conducted before and after the trainings. Data from two participants are missing, and therefore an analysis of 56 participants is presented in Table 2, which shows the average pre- & post-test scores for individual questions.

**Table 2.** Pre- and post-test results

Questions	Pre-test		Post-test		*p* Value
	No	%	No	%	
1. Mentally ill usually cannot make decisions	4	7	8	14	0.344
2. Mentally ill are best cared for in mental hospitals	22	39	39	70	0.009
3. All depression should be treated by antidepressants	36	64	38	68	1.000
4. Anti-depressants are addictive	33	59	50	89	0.002
5. Benzodiazepines are addictive	43	77	54	96	0.013
6. Mental disorders are common in children and adolescents	26	46	41	73	0.017
7. Acute seizures, I/M diazepam is the treatment of choice	31	55	38	68	0.265
8. Depression in a mother may lead to developmental delay in the child	43	77	50	89	0.118
9. For a child with over activity, medication is usually needed	27	48	41	73	0.007
10. Vitamin injections should be routinely used for somatic complaints	35	63	43	77	0.185
11. Asking about suicidal thoughts increases risk of suicide	19	34	33	59	0.016
12. Symptoms of depression	34	61	48	86	0.007
13. Treatment with anti-depressants	40	71	43	77	0.701
14. Psycho-social advice for depression	52	93	54	96	0.687
15. Diagnosis of ‘hearing voices’	42	75	53	95	0.006
16. Management of acute psychosis	30	54	28	50	0.851
17. Diagnosis of epilepsy	21	38	49	88	<0.001
18. Anti-epileptic medication	25	45	53	95	<0.001
19. Suicidal attempt	46	82	52	93	0.267
20. Drug dependence	47	84	53	95	0.109
21. Behavioral management of children	50	89	47	84	0.424
22. Managing developmental delay	36	64	47	84	0.027
23. Managing difficult behavior in adolescents	41	73	44	79	0.629
24. Motivation and treating drug dependence	40	71	46	82	0.238
25. Pharmacological treatments of mental illness	41	73	39	70	0.690

The first 11 questions were True/False and the rest were ‘choose the best answer’. Two questions from each category were changed. All four questions that were changed, related to dementia and alcohol dependence. Instead, two questions [Questions (Q) 4 & 5] were added on the dependence potential of anti-depressants and benzodiazepines in the True/False section. Similarly, two questions (Q 20 & 21) about drug dependence and the behavioral management of children were added to the second category.

The results of the analysis suggested encouraging levels of baseline knowledge, as more than 50% scored correctly on the pre-test questions. The exceptions were questions related to general principals of care (Q 1 & 2); child and adolescent mental health (Q 6 & 9); and risk of suicide (Q 11). There were four questions (Q 14, 19–21) where more than 80% scored correctly in the pre-test, hence may not be sensitive to measure impact of training.

The post-test evaluation demonstrates significant improvement in general physicians’ knowledge related to Epilepsy (Q 17 & 18); Psychosis (Q 15); common misconceptions about addictive potential of anti-depressants and benzodiazepines (Q 4 & 5); as well as acquisition of new information regarding child and adolescent mental health (Q 6, 9 & 22).

There were three questions (Q 1, 11 & 16) where at least a third of participants were unable to answer correctly, even after training. These related to: (a) ability of mentally ill to make decisions, (b) checking about suicidal thoughts and (c) management of psychosis.

The mean pre- and post-test scores of the participants were 15.43, 62% (*p* value 0.000, s.d. 4.05) and 19.48, 78% (*p* value 0.000, s.d. 3.13) respectively, which showed significant improvement.

Table 3 shows a summary of the feedback from the participants.

**Table 3.** Feedback from the participants

Question	Summary of responses
1. What was useful?	Increased basic knowledge about common mental disorders. Selection of modules was relevant to clinical needs. Training manual is precise and easy to understand. Information about the risk of dependence with benzodiazepines was very helpful. Group discussions and role-plays were most effective method of teaching. Training has helped in recognition, initial management and referral of common mental disorders
2. What was not useful?	Duration of training was short. Logistics/refreshments should be improved. Heating arrangements should be made
3. Any suggestions?	These workshops should be more frequent. Duration of workshop should be increased to at least three days. Continuous supervision/feedback of the participants in their workplaces would be very useful. Guidelines to manage a violent/aggressive patient should be included

## Discussion

There is growing evidence that mhGAP guidelines can be used to successfully train non-specialized health workers in resource-limited settings to recognize and treat common mental illnesses. According to the WHO (2013b), many developing countries like Libya, Jordan, Iraq, Afghanistan and Egypt are at various stages on the path to implement mhGAP guidelines. The same newsletter also mentioned that these guidelines were used to support Syrian refugees in the Kurdistan, Iraq region in 2013 and that Somalia chose an innovative strategy to integrate mhGAP-IG training into medical undergraduate curricula. In Lebanon, trainees showed an average of 12–25% improvement in knowledge, and 85% doctors and 91% nurses met minimum competency standards (Hijazi *et al.* 2011). The mhGAP training in Kashmir also reported improvement in knowledge related to mental healthcare. Similarly in Ethiopia and Nigeria, results show an increase in knowledge, access to healthcare and service utilization (Andrea, 2014; Gureje *et al.* 2015). These experiences reinforce the need to involve local mental health services, conduct refresher trainings and focus on offering sustainable services.

Our mhGAP intervention was the first of its kind following a humanitarian crisis for an internally displaced population in a high security zone in Pakistan. The training was very much part of an overall MHPSS response where monthly mental health camps were held (Humayun *et al.* 2016). These camps helped identify relevant & culturally sensitive training needs and the curriculum was accordingly adapted as per recommendations (Patel, 2013). Furthermore, the selection of priority disorders for the curriculum was guided by fieldwork, as opposed to discussion based curriculum described, for example by Hussain & Hughes, 2013. Furthermore, in Kashmir, participants were divided into large groups of prescribers and non-prescribers to address the diversity of participants. But we found that the diversity amongst much smaller groups (~20 each group) was most helpful.

In the interest of time, it might have been more appropriate to conduct this training based on the mhGAP–HIG (Humanitarian Intervention Guide), which was released shortly afterwards (WHO & UNHCR, 2015).

A major strength of this initiative was that the trainers were not just an active part of the emergency mental health response but were qualified psychiatrists, in contrast to other reports where the facilitators were either external or were non-specialist physicians (Gureje *et al.* 2015). The collaborations between public health services (primary, secondary and tertiary levels) and humanitarian agencies lasted over a year during this initiative. As recommended, the aim was to strengthen the existing local resources at all levels of the healthcare system through the provision of services, training and supervision (Pérez-Sales *et al.* 2011; Epping-Jordan *et al.* 2015). The intervention helped in engaging local services and provided an impetus for setting up a mental health agenda in the region. It was encouraging that during this initiative, the humanitarian forum formed the first ever MHPSS taskforce in the province (Humanitarian Response, 2015`).

In developing a curriculum, our main challenge was to introduce concepts of psychological medicine and psychotherapeutic interventions to an audience trained in a purely biomedical model. Like noted by Hijazi *et al.* 2011 mental healthcare provided through primary healthcare in Pakistan is also limited to the prescription of psychiatric medicines. Initially the physicians seemed reluctant to invest in psychological interventions fearing that these would be inappropriately demanding in terms of their clinical time and expertise. For this reason, and in order to engage them, we abridged these as ‘counseling’ without distinguishing among individual psychotherapeutic techniques. Our focus on the management of both stress-related and depressive disorders included all components of Problem Management Plus, now described as a trans-diagnostic psychological intervention (Rahman *et al.* 2016). Another challenge was the introduction of behavioral management for children with disabilities where we had to simplify the principles, use clinical examples, and emphasize the harm caused by psychotropic drug prescriptions for children – an unfortunately common practice. Additionally, the training consistently highlighted the need for psycho-education and the engagement of families in developing treatment plans.

For content, we added guidelines to manage stress and related disorders and excluded alcohol and dementia to make trainings more relevant to the context. In a similar setting, the Kashmir experience also excluded alcohol and dementia from their curriculum of priority disorders and highlighted the need to include stress-related disorders.

Pre-test scores showed clear gaps in the understanding of physicians in recognizing and managing common mental disorders. Some important findings regarding gaps in knowledge are discussed here:

There were significant misconceptions about psychotropic drugs: a third of doctors believed that antidepressants are addictive, while nearly 80% thought that benzodiazepines are not addictive. This likely explains the practice of fewer antidepressant prescriptions compared to benzodiazepines for the treatment of depression in other settings (Nakao *et al.* 2007). These findings are similar to another study in the country, which revealed that benzodiazepines were the most recognized and prescribed category of medication (75.3%) by general physicians and only a few were familiar with selective serotonin reuptake inhibitors (35.1%) or tricyclic antidepressants (20.2%) (Naqvi *et al.* 2012).

It was worrying to note that two-thirds of participants believed that inquiring about self-harm increased the risk of suicide. Despite emphasizing the need and even conducting a role-play specifically to assess the risk of self-harm, only 59% participants responded correctly afterwards.

The need to focus more on the much-neglected area of mental health of children & adolescents was evident. It is known that child psychiatry training can be successfully implemented for non-specialists in low-resource settings (Tesfaye *et al.* 2014). A conceptual gap also existed for substance abuse, which was largely viewed as a moral issue. Just a few considered it a ‘disorder’ with little awareness about evidence based treatment guidelines. From the feedback on our module on psychosis, we realized that principles of rapid tranquilization should also have been included to help deal with emergencies in the community. Surprisingly, more than half of the participants scored incorrectly on questions related to the diagnosis and drug treatment of epilepsy.

The results also highlighted some gaps in understanding ‘general principles of care’, which focus on changing attitudes and responding to the needs of the mentally ill in a respecting and non-judgmental manner. For example, the majority continued to believe that mentally ill patients cannot make their own decisions, and a third still maintained that mentally ill patients are best cared for in mental hospitals, even after training. But encouragingly, toward the end of training workshops, participants recognized that their existing knowledge and attitudes might well be a reflection of prevailing stigma related to mental health problems in Pakistan. A separate session on general principles of care should be considered in future trainings.

The logistic difficulties of undertaking this initiative have been described elsewhere in detail (Humayun *et al.* 2016). In a system where mental health needs are not even recognized as a priority, the district health department was also largely unaware and unobliging. This barrier was overcome through the strong influence of the military authorities that had administrative control of the crisis zone. The second important partnership was with the local department of psychiatry that was aware of the need to engage primary care physicians but struggling to redefine its role beyond hospital based clinical work.

The limitations of this initiative are best discussed in the context of an underresourced primary healthcare system in Pakistan (National Health Vision 2015–2025, 2016). It is also important to note that this was neither a government nor an institutional led initiative, but was coordinated by a small group of volunteer mental health professionals. Unfortunately, IMC could not organize refresher trainings as part of this initiative, but we did provide hands-on supervision for 6 months at the camps and the department of psychiatry undertook to arrange refresher trainings to follow up as well. The main limitation of this study is that it does not evaluate the impact of the training on recognition and management of common mental disorders or any influence on referral patterns. As a result, and in view of the absence of an effective health information system, we do not have a measure of a quantitative outcome of these training, although anecdotal reports from various sources suggested that the target population continued to utilize mental healthcare in the region. In addition, we found it challenging to engage an inclusive team (including nurses, psychologists and social workers) in the primary care since most healthcare interventions are usually left to doctors. The psychosocial staff in the field was from humanitarian agencies, which complemented services in the aftermath of the crisis, but which might not sustain in the longer term. Another limitation was a severe dearth of permanent community services, thereby restricting opportunities for referral linkages. As a result of these limitations, there is a serious concern about integrating mental health services meaningfully into a resource limited primary care system in countries such as Pakistan.

## Conclusion

Although the crisis triggered by internal displacement provided an opportunity to take up this initiative, there is a pressing need for sustainable capacity building at the provincial level in Pakistan. It is only recently that one of the authors (AH) was part of a consultancy where the government of KP partnered with international agencies to assess and strengthen institutional and community-based mechanisms to respond effectively to the medium- to long-term MHPSS needs in the province. Siriwardhana *et al.* (2016) have demonstrated the feasibility of training primary care practitioners to promote the integration of mental health into primary care for post-conflict populations in the region. This may well be a way forward for strengthening mental health services in post-conflict areas in Pakistan as well. Quite encouragingly, at the time of the final submission of this report, a pilot project implementing mhGAP training in primary healthcare has been initiated across five districts in Pakistan. Persistent efforts for robust advocacy and collaboration at policy, training and service levels are much needed. In order to overcome the dearth of resources, collaborations between academic institutions and humanitarian agencies would greatly help in disseminating these guidelines.
